# Scalable Passaging of Adherent Human Pluripotent Stem Cells

**DOI:** 10.1371/journal.pone.0088012

**Published:** 2014-01-30

**Authors:** Ying Nie, Patrick Walsh, Diana L. Clarke, Jon A. Rowley, Thomas Fellner

**Affiliations:** Cell Therapy Development Services, Lonza-Walkersville, Inc., Walkersville, Maryland, United States of America; French Blood Institute, France

## Abstract

Current laboratory methods used to passage adherent human pluripotent stem cells (hPSCs) are labor intensive, result in reduced cell viability and are incompatible with larger scale production necessary for many clinical applications. To meet the current demand for hPSCs, we have developed a new non-enzymatic passaging method using sodium citrate. Sodium citrate, formulated as a hypertonic solution, gently and efficiently detaches adherent cultures of hPSCs as small multicellular aggregates with minimal manual intervention. These multicellular aggregates are easily and reproducibly recovered in calcium-containing medium, retain a high post-detachment cell viability of 97%±1% and readily attach to fresh substrates. Together, this significantly reduces the time required to expand hPSCs as high quality adherent cultures. Cells subcultured for 25 passages using this novel sodium citrate passaging solution exhibit characteristic hPSC morphology, high levels (>80%) of pluripotency markers OCT4, SSEA-4, TRA-1-60 andTRA-1-81, a normal G-banded karyotype and the ability to differentiate into cells representing all three germ layers, both *in vivo* and *in vitro*.

## Introduction

Advancing pluripotent stem cell research to clinical applications requires adapting laboratory-scale cultivation methods to less conventional and current Good Manufacturing Practices- (cGMP-) compliant platforms [1], [2]. As a first step, we set out to improve and simplify conventional methods used to subculture hPSCs. Current practices include the propagation of adherent colony cultures, as multicellular aggregates, using one or a combination of methods that include manual scraping, manual microdissection, enzymatic and non-enzymatic procedures to detach the cells from their matrix. Manual microdissection and scraping portions of selected colonies is labor intensive, and highly dependent on the proficiency of skilled technical personnel. Enzymatic and existing non-enzymatic methods are time-critical. Over-treatment of the cells with the detachment solution often results in the increased production of single cells [3]. Unfortunately, the continuous cultivation of adherent hPSCs from single cells [4] may play a role in promoting chromosomal abnormalities and genetic alterations in a hPSC population over time [5], [6]. Moreover, single cells produced during the subculture of hPSC colonies are susceptible to dissociation-induced apoptosis [7].

To define a new technology for the subcultivation of adherent hPSCs, several fundamental properties were reasoned to be critical for it to be considered a broadly useful and successful advance to meet the increasing demand, both in research and clinical applications. First, the technology must preserve the conventional redistribution of intact hPSC colonies as small multicellular aggregates. The subculture of hPSCs as multicellular colony aggregates sustains lateral intercellular contacts that are important in cell to cell communication. This reduces cell stress, minimizes spontaneous cell differentiation and promotes cell survival [8], [9]. Once plated, the aggregates must quickly re-establish themselves on fresh matrix as monolayer colonies with the characteristic morphology of undifferentiated hPSCs. Use of the technology must also enable easy and reproducible recovery of hPSCs from both conventional laboratory culture vessels and access-restricted multilayer flasks without mechanical intervention. Finally, the technology must maintain the pluripotent nature of the cells, their differentiation potential and have no adverse effect on the cell's viability or general genetic stability.

## Materials and Methods

### hPSC cultivation

The feeder-independent human embryonic stem cell (hESC) line, WA09, was obtained from the WiCell mTeSR™1 Bank and cultivated in either mTeSR™1 (Stem Cell Technologies, 05850) or adapted to StemPro® hESC SFM (Life Technologies, A1000701). Cell stocks were continuously maintained in feeder-independent conditions on hESC-Qualified Matrigel™ (Becton Dickinson, 354277) in a humidified, 37°C cell incubator equilibrated with 5% CO_2_. Later, an additional feeder-independent hESC line, WA07 (WiCell mTeSR™1 Bank) and two human induced pluripotent stem cell (hiPSC) lines, hiPSC18R and hiPSC19K (Lonza–Walkersville), were also cultivated continuously in the hypertonic citrate solution to determine if additional hPSC lines could also maintain their pluripotency. Both hiPSC lines were generated from human cord blood CD34+ cells.

### Hypertonic sodium citrate solution

The 1 mM hypertonic sodium citrate solution was prepared by dissolving 0.2941 grams of sodium citrate dihydrate in 800 mls of water containing 22.975 grams of potassium chloride. The solution volume was then increased to one liter with water. The final osmolality of the solution was adjusted to 570 mOsm/kg using potassium chloride. The osmolality was verified using an Osmometer (Model 3D3, Advanced Instruments). Additional 1 mM sodium citrate solutions of varying osmolalities were prepared in a similar manner with incremental adjustments made to the amount of potassium chloride present in the initial one liter solution.

### Optimization of citrate detachment

WA09 hESCs were cultivated in tissue culture-treated six-well plates coated with hESC-Qualified Matrigel™ (Becton Dickinson 354277). Once cells reached confluence, the cell culture medium was aspirated and the cells were rinsed once with 1 ml 1× Dulbecco's Phosphate Buffered Saline without calcium and magnesium (DPBS^−/−^; Lonza Bioscience 17-512F). The DPBS^−/−^ was immediately removed. One ml of one 1 mM citrate solution representing the range of osmolalities tested (270, 420, 570, 720 mOsmol/kg), was then added to each well of one six-well plate. Three replicate wells in each plate were incubated in the citrate solution at room temperature for 10 minutes. The remaining three replicate wells were incubated for 20 minutes. The citrate solution was subsequently removed and 1 ml of complete culture medium added to each well. To mimic the delay in cell recovery from access-restricted multilayer vessels, the medium was left on the cells for 5 minutes. Cells were then released into the medium by gentle tapping of the plate. The detached cell aggregates were sampled from each well and imaged to quantify the distribution of the aggregate size. The percentage of the detached culture present as single cells or aggregates of less than four cells was calculated and plotted against the osmolality of the citrate solution.

### Image analysis to determine multicellular aggregate size

Dissociated cultures were sampled and imaged live. Colony aggregates were assumed to be planar and quantified with respect to fragment surface area. Measurements were recorded from six images for each sample using NIS-Elements BR 3.22.08 imaging software (Nikon). For each sample, recorded fragment surface areas were categorized into size ranges. Surface areas of fragments smaller than a 4-cell aggregate were added and normalized to the total surface area measured for each sample. These data were plotted against the osmolality of the citrate solution.

### Hypertonic citrate colony detachment

WA09 hESCs were adapted and cultivated in tissue culture-treated six-well plates coated with hESC-Qualified Matrigel™. Once the cells reached confluence, the cell culture medium was aspirated and the cells were rinsed once with 1 ml 1× DPBS^−/−^ (Lonza Bioscience 17-512F). The DPBS^−/−^ was immediately removed and 1 ml of the hypertonic citrate solution added to the wells. The solution was allowed to incubate on the cells for 5 to 15 minutes at room temperature. Cells were monitored under the microscope until gaps appeared between them and the edges of the colonies began to shrink or lift from the well surface. The hypertonic citrate solution was then removed, and one or more milliliters of complete culture medium released as a stream from a pipet to gently rinse the colony aggregates from the well surface. Cells were collected individually from replicate wells (n = 3) directly into sterile conical tubes, counted, and seeded into new matrix-coated plates.

### Enzymatic colony detachment

WA09 hESCs were cultivated in tissue culture-treated six-well plates coated with hESC-Qualified Matrigel™ (Becton Dickinson 354277). Once cells reached confluence, the cell culture medium was aspirated and the cells were rinsed once with 2 ml DMEM/F12. Following removal of the medium, either 1 ml of Dispase (Life Technologies 17105-041, reconstituted at 1 mg/ml in DMEM/F12) or 1 ml of Collagenase Type IV (Life Technologies 17104-019, reconstituted at 1 mg/ml in DMEM/F12) was added to each well and allowed to incubate on the cells for 5 minutes at 37°C. Enzyme was then removed, fresh culture medium added, and the cells scraped from the plates with a 5 ml serological glass pipette. Detached cells were collected individually from replicate wells (n = 3), collected by centrifugation at 200× g, the supernatant aspirated and fresh medium added to the cells prior to plating on fresh matrix.

### Manual colony detachment

WA09 hESCs were cultivated in tissue culture-treated six-well plates coated with hESC-Qualified Matrigel™. Once the cells reached confluence, the cell culture medium was aspirated and the cells were rinsed once with 2 ml DMEM/F12. Following removal of the medium, 1 ml culture medium was added to the well and portions of the colonies manually scraped from the well surface using a 5 ml glass serological pipette. Detached colony aggregates were collected individually from replicate wells (n = 3), collected by centrifugation at 200× g, the supernatant aspirated and fresh culture medium added to the cells prior to plating on fresh matrix.

### Cell expansion

WA09 hESCs were cultivated in either mTeSR™1 or StemPro® hESC SFM on hESC-Qualified Matrigel™. Cells were initially seeded in triplicate on fresh Matrigel™-coated Tissue Culture-treated six-well plates at 2×10^5^ viable cells per well (P0). This same seeding density was maintained for each passage. Culture medium was exchanged every day. When the cell colonies reached confluence, the cells were detached from the wells (P1) with conventional scraping, Collagenase Type IV (1 mg/ml), Dispase (1 mg/ml) or the hypertonic citrate solution (1 mM Sodium Citrate at 570 mOsmol/kg). Cell number and viability were determined on each well using a Nucleocounter NC-100 according to the manufacturer-recommended protocol. The total number of viable cells present at the time of each passage was determined and used to re-seed the cells at 2×10^5^ for each subsequent passage. Since it was not necessary to seed all the detached cells, the total number of viable cells was calculated for P1 to P5 and continuously extrapolated over time to predict the total number of viable hESCs that could be generated.

### Embryoid body differentiation

Confluent cultures of WA09 hESC colonies were dissociated with hypertonic citrate solution. Cell aggregates were suspended in EB formation medium (50% DMEM/F12 (Life Technologies, 11330-032) 50% mTeSR™1 or StemPro® hESC SFM containing 10 µM Rock Inhibitor Y27632 (Millipore, SCM075)) and allowed to settle by gravity in a conical tube. The supernatant was subsequently removed and the cells suspended in fresh EB medium. Cell aggregates were then seeded at a split ratio of 1∶1 on Ultra Low Attachment (Corning, YO-01835-24) plates and returned to the incubator for 12 to 24 hours. Once large cell aggregates formed they were collected into a conical tube and allowed to settle by gravity. The medium was then removed and replaced with differentiation medium (80% DMEM High Glucose (Life Technologies, 11965-092), 20% defined fetal bovine serum (Hyclone, SH30070.03), 1× non-essential amino acids (Life Technologies, 11140-050), 2 mM L-glutamine (Life Technologies, 35050-061) and 55 µM (final) β-Mercaptoethanol (Life Technologies, 21985-023)). The aggregates were placed back on Ultra Low Attachment plates using a split ratio of 1∶1 at 0.4 ml differentiation medium/cm^2^. Culture medium was exchanged every second day for six days. On the seventh day, the EBs were placed on gelatin-coated plates (EmbryoMax® ES Cell Qualified Gelatin Solution (Millipore, ES006-B)) at approximately 10 EBs/cm^2^. The EBs were allowed to attach undisturbed for 2 days. The differentiation medium was exchanged after the second day and every other day afterward with 0.4 ml/cm^2^ differentiation medium. Cultures were prepared for immunocytochemistry on day 14.

### Immunocytochemistry

Culture medium was aspirated, and the cultures washed twice with 1× Dulbecco's Phosphate Buffered Saline (DPBS, Lonza Biosciences 17-513F). Cells were fixed in 1× DPBS containing 4% PFA (Electron Microscopy Sciences, 15710) for 20 minutes, then permeabilized for 30 minutes in 1× DPBS containing 0.1% Triton X-100 (Sigma-Aldrich, T9284) for 30 minutes. Cells were subsequently washed twice with PBS-T (0.2% Tween-20 (Sigma-Aldrich, P9416) in 1× DPBS). Cells were then placed in blocking buffer (PBS-T containing 10% blocking serum) for two hours prior to the addition of antibody.

For embryoid body (EB) staining, primary antibodies detecting alpha-1 Fetoprotein (Abcam, ab3980; 1∶200), beta tubulin III (Millipore, MAB1637; 1∶400) and Smooth Muscle Actin (DAKO, M0851; 1∶400) were added to blocked cultures and incubated overnight. Cultures were washed twice in blocking buffer, and a secondary antibody, Alexa 488-conjugated goat anti-mouse IgG (Life Technologies, A11001; 1∶400) was added and incubated on the cells for at least 2 hours. Cultures were then rinsed three times (10 minutes each) in 1× DPBS prior to being prepared for microscopic analysis.

Similar to EB staining, primary antibodies raised against pluripotency-associated antigens detecting OCT4 (Abcam, ab19857; 1∶350), Sox2 (Abcam, ab97959; 1∶100) and Nanog (Abcam, ab21624; 1∶50) were used in combination with secondary antibody Alexa 488-conjugated Donkey anti-rabbit IgG (Jackson ImmunoResearch, 711545152; 1∶200). Antibody detecting SSEA4 (Millipore MAB4304; 1∶100) was used in combination with the secondary antibody, DyLight 594-conjugated Donkey anti-mouse IgG (Jackson ImmunoResearch, 715515150; 1∶200); and antibodies detecting TRA-1-60 (Millipore, MAB4360; 1∶100) and TRA-1-81 (StemGent, 090011 1∶100) were used in combination with the secondary antibody DyLight 594-conjugated Donkey anti-mouse IgM (Jackson ImmunoResearch, 715505140; 1∶200). All cells were counterstained with 1 µg/ml DAPI (Sigma-Aldrich, D9542) in 1× DPBS to fluorescently label cell nuclei. All fluorescence detection was visualized using an EVOS FL all-in-one microscope equipped with software version 17625.

### Flow cytometry

hESCs, cultivated in either mTeSR™1 or StemPro® hESC SFM for over 25 cell passages, were dissociated into a single-cell suspension using a solution of TrypLE (Life Technologies, A12A59) containing 2% chick serum (Sigma-Aldrich, C5405). Cells were fixed and permeabilized for intracellular staining with the Cytofix/Cytoperm Kit (Becton Dickinson, 554714) following the manufacturer's recommended protocol. Permeabilized cells were incubated with PE-conjugated anti-OCT3/4 (R&D Systems IC1759P; 1∶50,000) or respective PE-conjugated anti-IgG isotype control.

Extracellular antigens were detected on unfixed cells stained with PE-conjugated antigen-specific antibodies and respective isotypes: anti-TRA-1-60 (Becton Dickinson, 560193; 1∶50,000), anti-TRA-1-81 (Becton Dickinson, 560161; 1∶50,000), anti-IgG3 isotype (Becton Dickinson, 559926; 1∶200,000); anti-SSEA4 (Becton Dickinson, 560128; 1∶50,000) and anti-IgM isotype (Becton Dickinson, 555584; 1∶50,000). Samples were processed through a FACS Calibur (Becton Dickinson) flow cytometer. Data were acquired using CellQuest Pro 5.2.1 and analyzed with Flowjo 7.6 software.

### Teratoma formation

WA09 hESC colonies, cultured in either mTeSR™1 or StemPro® hESC SFM for over 25 cell passages, were dissociated using the hypertonic citrate solution. Cells were counted, distributed into tubes, and pelleted. 1.33×10^6^ cells were suspended in hESC-Qualified Matrigel™. The cell suspension was injected intramuscularly into the right hind leg flank of six week old SCID/Beige mice. Three mice were injected per condition. The mice were observed daily, and the tumor measured twice a week by veterinary services staff and recorded. The tumors were allowed to grow to 2.0 cm in diameter. Once the tumor reached this measurement, the animal was euthanized and the tumor excised, embedded in paraffin and sectioned in 4 µm serial sections. Slides with representative cell types were stained with hemotoxylin and eosin. Pathology was also performed on control tissues derived from the non-injected legs of these mice.

### Animal Ethics Statement

The SCID/Beige mice used to produce the experimental teratomas in this study were housed in Lonza Walkersville, Inc.'s AAALAC accredited (Association for the Assessment and Accreditation of Laboratory Animal Care) animal facility and were cared for in accordance with the principles outlined in the ILAR Guide for the Care and Use of Laboratory Animals. The animal use was approved by the (Lonza Walkersville) Institutional Animal Care and Use Committee in accordance with the USDA Animal Welfare Act.

### Karyotype analysis

To determine if any large genetic abnormalities were present after the hPSCs were continuously passaged with hypertonic citrate, three independent WA09 cultures were continuously cultivated exclusively in either mTeSR™1 or StemPro® using the 570 mOsmol/kg citrate solution. Exponentially proliferating cultures from each independent culture were prepared on or after passage number twenty five (mTeSR™1 (P27); StemPro® (P25)) and sent to Cell Line Genetics (Madison, Wisconsin). Cytogenetic analysis was performed on a minimum of twenty G-banded metaphase cells for each independent sample. All six samples (3 using mTeSR™1 and 3 using StemPro®) were normal based on this analysis. Comparative G-banding analysis of the additional hPSC lines, the WA07 hESC line (P40) and two iPSC lines, hiPSC18R (P30) and hiPSC19K (P30), were also found to be karyotypically normal.

### Statistical analysis

All statistical calculations including the mean, standard deviation and standard error of the mean (SEM) were calculated using Microsoft Excel. Statistical significance (P-value) was calculated manually using a type-3 T-test.

## Results

### Identification and optimization of sodium citrate as a passaging reagent

We initially evaluated ethylenediaminetetraacetic acid (EDTA) as an alternative passaging solution for scalable cultivation of hPSCs. A 0.5 mM solution of EDTA was recently reported as a new non-enzymatic hPSC passaging solution that yields multicellular aggregates with high post-detachment cell viability [3]. To test EDTA's performance limits for scalable production of hPSCs, WA09 hESCs were cultivated in access-restricted multi-layer cell culture flasks. Cells were treated with 0.5 mM EDTA until the cells began to round up from the surface and gaps began to develop between cells within the colonies. Fresh culture medium was added to the multi-layer flask to collect the cells. The flask was gently agitated, by tapping, to release the aggregates from their substrate. However, before the cells could be recovered from the flask, a substantial portion of the cells quickly re-attached back to the culture surface. Attempts to recover more cells by using longer treatment times or calcium-free medium to collect the cells only resulted in further dissociation of the multicellular aggregates to single cells.

EDTA chelates divalent cations and promotes cell dissociation and attachment by sequestering calcium and magnesium. To define a passaging solution that would perform better in multi-layer culture, we selected another non-enzymatic chelating agent, sodium citrate, and optimized its formulation to meet the critical attributes originally identified. Chemically, sodium citrate is a much milder chelating reagent [10], a property that could potentially reduce or eliminate the critical time constraints observed with EDTA detachment and improve the proportion of cells recovered as multicellular aggregates. Relatively high concentrations of sodium citrate, 10 to 15 mM, have long been used for non-enzymatic cell dissociation in flow cytometry where the generation of single cells and preservation of cell surface markers is critical for cell sorting. By simply lowering the concentration of sodium citrate to 1 mM, WA09 cells could be detached as very small multicellular aggregates and single cells over extended treatment times (data not shown). However, by incrementally increasing the osmolality of the 1 mM sodium citrate solution, we found that it not only reduced the number of single cells generated but it also allowed control over the size of the detached multicellular aggregates produced.

**Figure 1:**
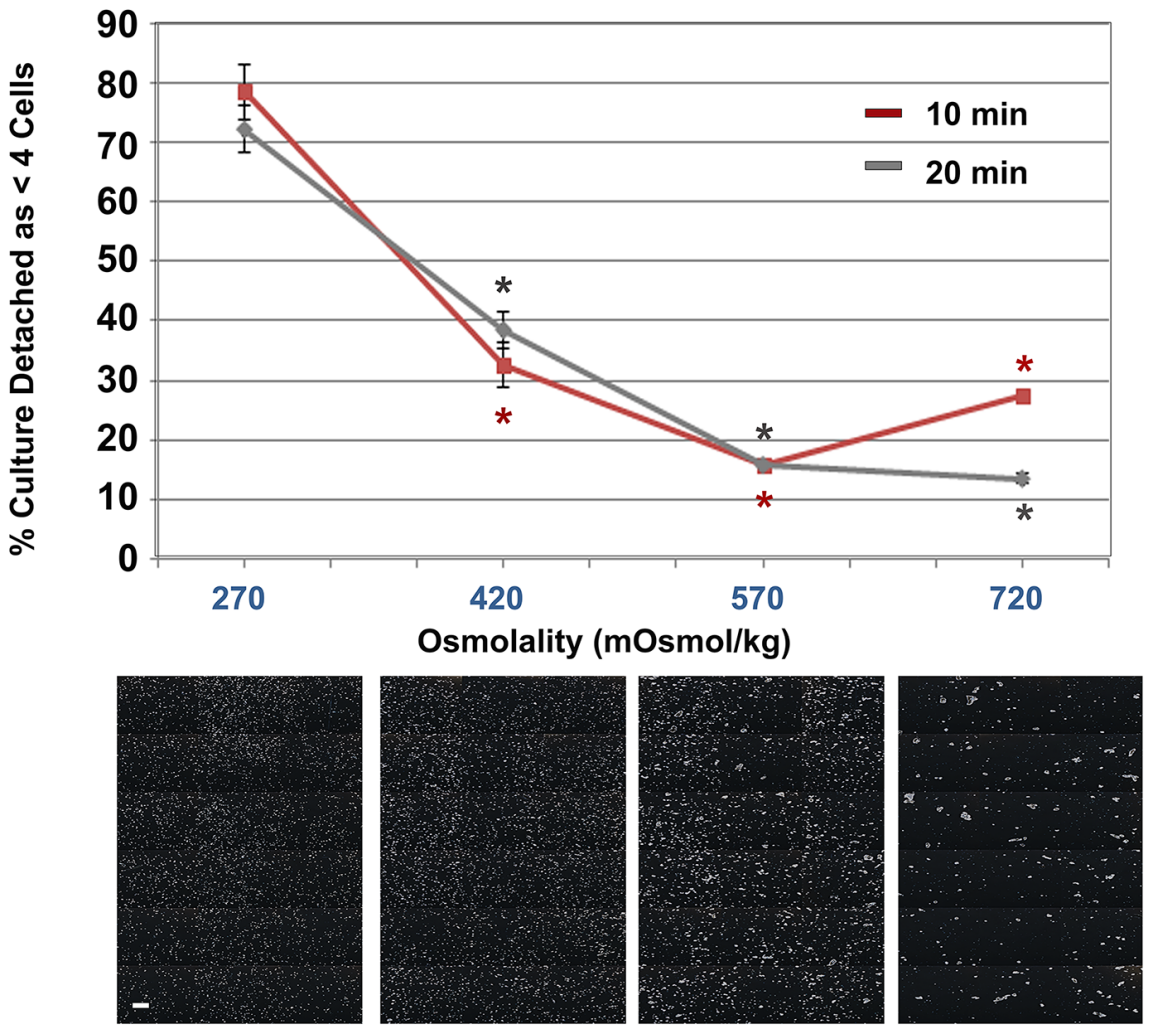
Subculture of WA09 hESCs with hypertonic citrate solution supports detachment of colonies as multicellular aggregates. Confluent WA09 hESC cultures maintained in mTeSR™1 on Matrigel™ were dissociated in a 1 mM sodium citrate solution at increasing osmolalities. Using image analysis software, six images of detached cells from each of three independent cultures (n = 3) were taken after 10 or 20 minutes of treatment with the citrate solutions. These images were quantified to assess the size of the multicellular colony aggregates produced. Error bars indicate standard error of the mean. Representative brightfield images of detached hESC multicellular colony aggregates, after 20 minutes of treatment, are shown below each osmolality. Using the isotonic osmolality (270 mOsmol/kg) as a reference, all other osmolalities exhibited statistically fewer single cells and aggregates less than four cells (P<0.05); scale bar: 100 µm.

To determine the optimal osmolality for the subcultivation of hESCs using a 1 mM hypertonic citrate solution, we compared the isotonic formulation (270 mOsmol/kg) with three additional solutions of increasing osmolality; 420, 570, and 720 mOsmol/kg. Confluent cultures of WA09 cells were treated with each of the four solutions and the detached aggregates imaged and analyzed after 10 or 20 minutes of treatment (Fig. 1). Photographs taken after 20 minute incubation in each osmolality are shown below the graph to illustrate the stable size distribution of the multicellular aggregates. Figure 1 illustrates that there is a clear decrease in colony detachment as single cells and an increase in the size of the detached cell aggregates as the osmolality increases between 270 and 720 mOsmol/kg. Quantification of aggregate size found that after 20 minutes of treatment, more than 72% of the total detached cells were present as single cells or very small aggregates (less than 4 cells) at 270 mOsmol/kg. Comparatively, when the sodium citrate solution was adjusted to 570 mOsmol/kg, only 16% of the detached cells were present as single cells or very small aggregates. Using the isotonic formulation (270 mOsmol/kg) as a reference, all other osmolalities tested produced larger cell aggregates and fewer single cells (P<0.05). These larger colony aggregates easily rinse from plates and multi-layer flasks without the need to scrape cells from the surface. When seeded on plates and cultivated, the multicellular aggregates produced at 570 mOsmol/kg attach readily, spread and proliferate as small monolayer colonies. While larger colony aggregates were produced at 720 mOsmol/kg, these cells did not spread well on Matrigel™ as a monolayer. Portions of the colonies remained as undesirable clumps of cells that often differentiated.

**Figure 2:**
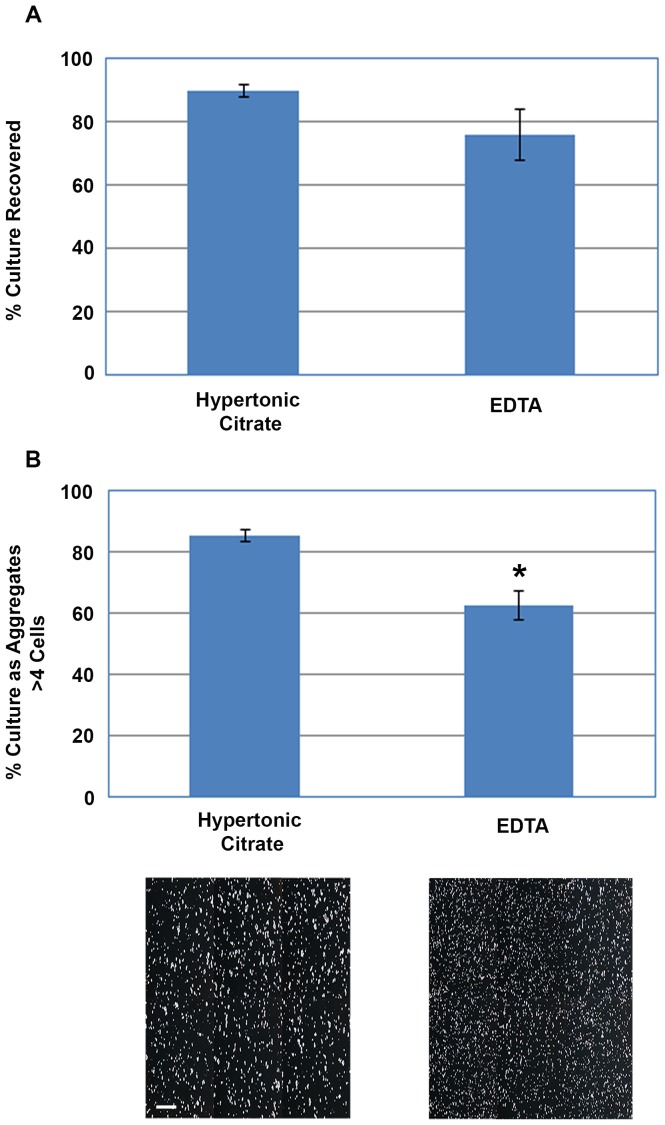
Comparison of hypertonic citrate solution with EDTA in the detachment of hPSCs as multicellular aggregates. (A) Percentage of WA09 cells recovered from multi-layer flasks using either the Hypertonic Citrate Solution (1 mM; 570 mOsmols/kg) or EDTA (0.5 mM); P>0.05. (B) Size quantification and brightfield images of the hESC aggregates obtained after a 20 minute treatment with hypertonic citrate or EDTA show the cell aggregates collected using the hypertonic citrate solution were larger and contained fewer single cells and very small aggregates (P<0.05). Error bars indicate standard error of the mean. All conditions, n = 3; scale bar: 200 µm.

The optimized hypertonic sodium citrate formulation (1 mM, 570 mOsmol/kg) was then directly compared with the 0.5 mM EDTA to determine if the hypertonic sodium citrate formulation could improve the collection of hPSCs from access-restricted multi-layer flasks. The hypertonic sodium citrate solution reproducibly recovered 90±2% of the cells while only 76±8% of the cells were recovered using EDTA (Fig. 2A). While this difference was not statistically significant (P>0.05), the variability observed using EDTA makes its use unsatisfactory for cGMP manufacturing where batch to batch processes must be highly reproducible. Moreover, the ability of both solutions to preserve the detached cells as multicellular aggregates over prolonged treatment was also compared (Fig. 2B). Cultures treated for 20 minutes with the hypertonic citrate solution produced aggregates that were larger than cultures similarly treated with EDTA. Quantification of these detached aggregates showed that the hypertonic citrate solution generated fewer single cells and very small aggregates (16%±1.9%) compared to the cultures detached with EDTA (37%±4.9%); P<0.05.

### Comparison of hypertonic sodium citrate to existing passaging technologies

**Figure 3:**
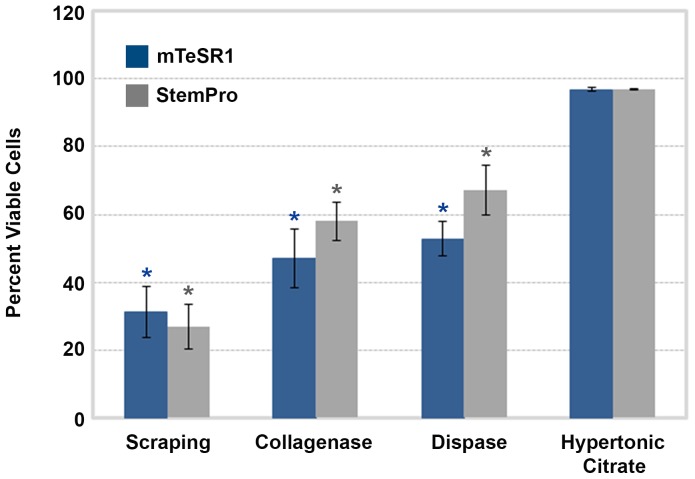
Comparison of WA09 cell post-detachment viabilities using conventional methods and the optimized hypertonic citrate solution. Using the hypertonic citrate solution as a reference, all other conventional detachment methods exhibited lower cell viability (P<0.05). Error bars indicate standard error of the mean. All conditions, n = 3.

Comparison of the post-detachment viability of hESCs treated with a 570 mOsmol/kg 1 mM sodium citrate solution with other conventional methods promoting multicellular detachment of hPSCs, further illustrates its effectiveness (Fig. 3). The hypertonic citrate solution reproducibly results in a post-detachment cell viability of 97%±1%. It is equally effective on cells cultivated in two defined hPSC cultivation media, StemPro® hESC SFM (StemPro®) or mTeSR™1. Conventional colony scraping, Collagenase IV and Dispase treatment exhibit significantly lower cell viabilities of 27%, 58% and 67% in StemPro® and 31%, 47% and 53% in mTeSR™1, respectively. Using the hypertonic citrate solution as the reference, all other conventional detachment methods generated significantly fewer viable cells (P<0.05), regardless of the medium utilized.

**Figure 4:**
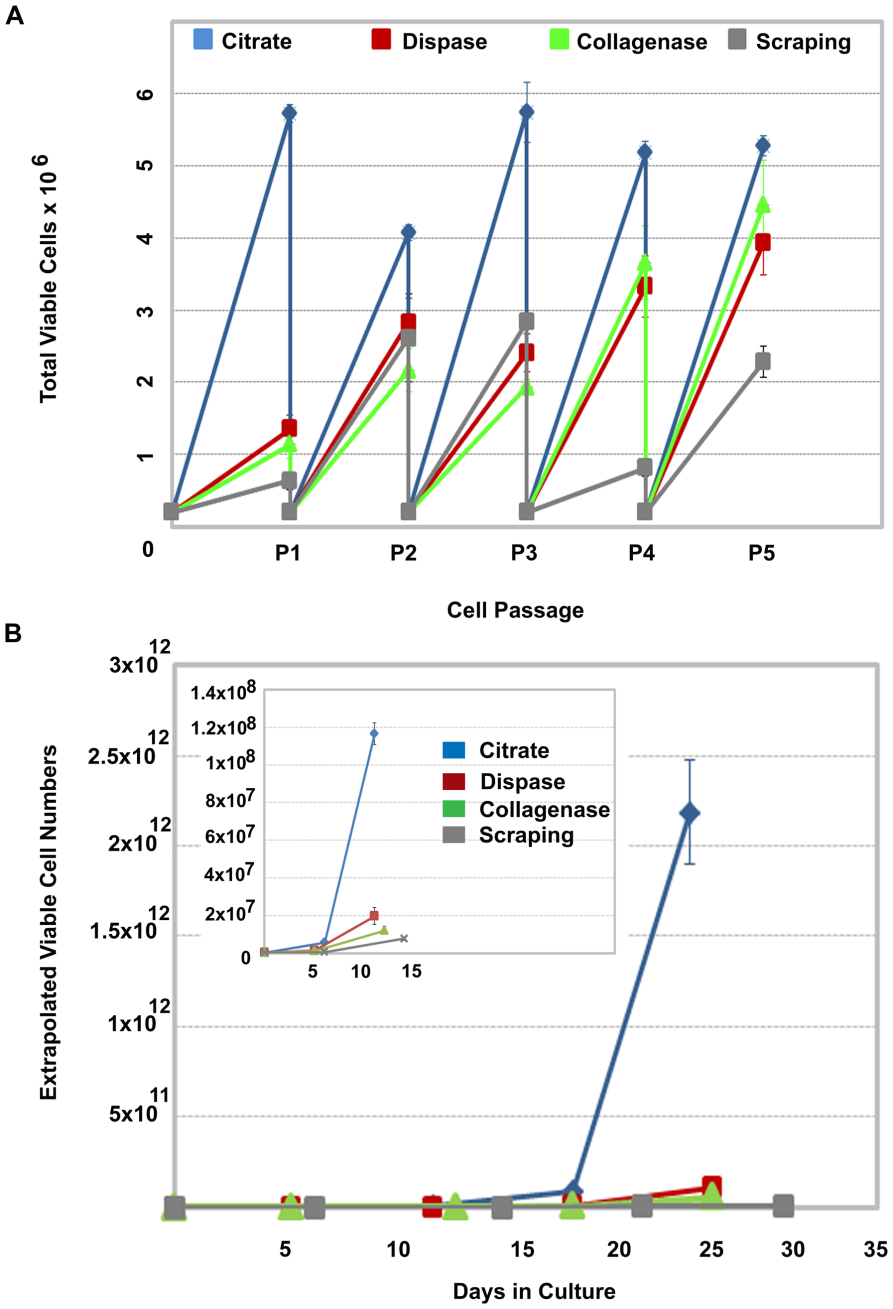
Post-detachment cell viability impacts the rate of cell expansion. (A) Comparison of cell numbers generated over 5 passages in mTeSR™1 on Matrigel™ using conventional colony scraping, Collagenase IV, Dispase or a 1 mM, 570 mOsmol/kg hypertonic sodium citrate solution to subculture the cells. Cell detachment methods were compared by continuously seeding 2×10^5^ viable cells per well in six-well plates to control for differences in post-detachment cell recovery. When the hESC colonies for each condition reached confluence, cells were passaged and the viable number of cells determined. Three replicate wells were then individually re-plated at 2×10^5^ and the process repeated. (B) The actual viable cell number determined at each passage was used to determine the total number of viable cells that would have been generated if all cells at each passage had been plated. Inset illustrates day 0 to day 15 with an expanded Y axis to illustrate the earlier passages. Error bars indicate standard error of the mean. All conditions, n = 3.

To evaluate the impact the 1 mM hypertonic citrate solution has on large-scale hPSC expansion on planar surfaces, we compared the total number of viable hESCs that were produced in mTeSR™1 over 5 passages using the hypertonic citrate solution, conventional colony scraping, Collagenase IV and Dispase treatment as agents to passage the cells. WA09 hESCs were continuously seeded at 2×10^5^ viable cells/well in six-well plates and allowed to proliferate until confluence before passaging. The total number of viable cells generated from 2×10^5^ viable cells at each passage using the different methods is shown in Figure 4A. As early as passage 5 (day 27), the total number of cells that would have been produced over this time period if all the cells generated at each passage were carried forward, exceeds 2×10^12^ using the hypertonic 1 mM citrate solution (Fig. 4B). To produce an equivalent number of cells using manual scraping, Collagenase IV or Dispase treatment would require approximately 97, 56 and 55 days, respectively (Table 1).

**Table 1:** Comparison of cell expansion using hypertonic citrate and conventional methods.

Reagent	Post-Dissociation Viability (mean)	Viable Cells Produced at Each Passage (mean)	Extrapolated Cell Number at P5	Days to Produce 2×10^12^ Cells
**Hypertonic Citrate Solution**	97±1%	5.2×10^6^±6%	2.32×10^12^	27
**Dispase**	53±5%	2.8×10^6^±16%	8.05×10^10^	55
**Collagenase**	47±9%	2.7×10^6^±23%	4.79×10^10^	56
**Scraping Only**	31±8%	1.8×10^6^±25%	4.97×10^9^	97

### Characterization of hESCs subcultured with hypertonic citrate

**Figure 5:**
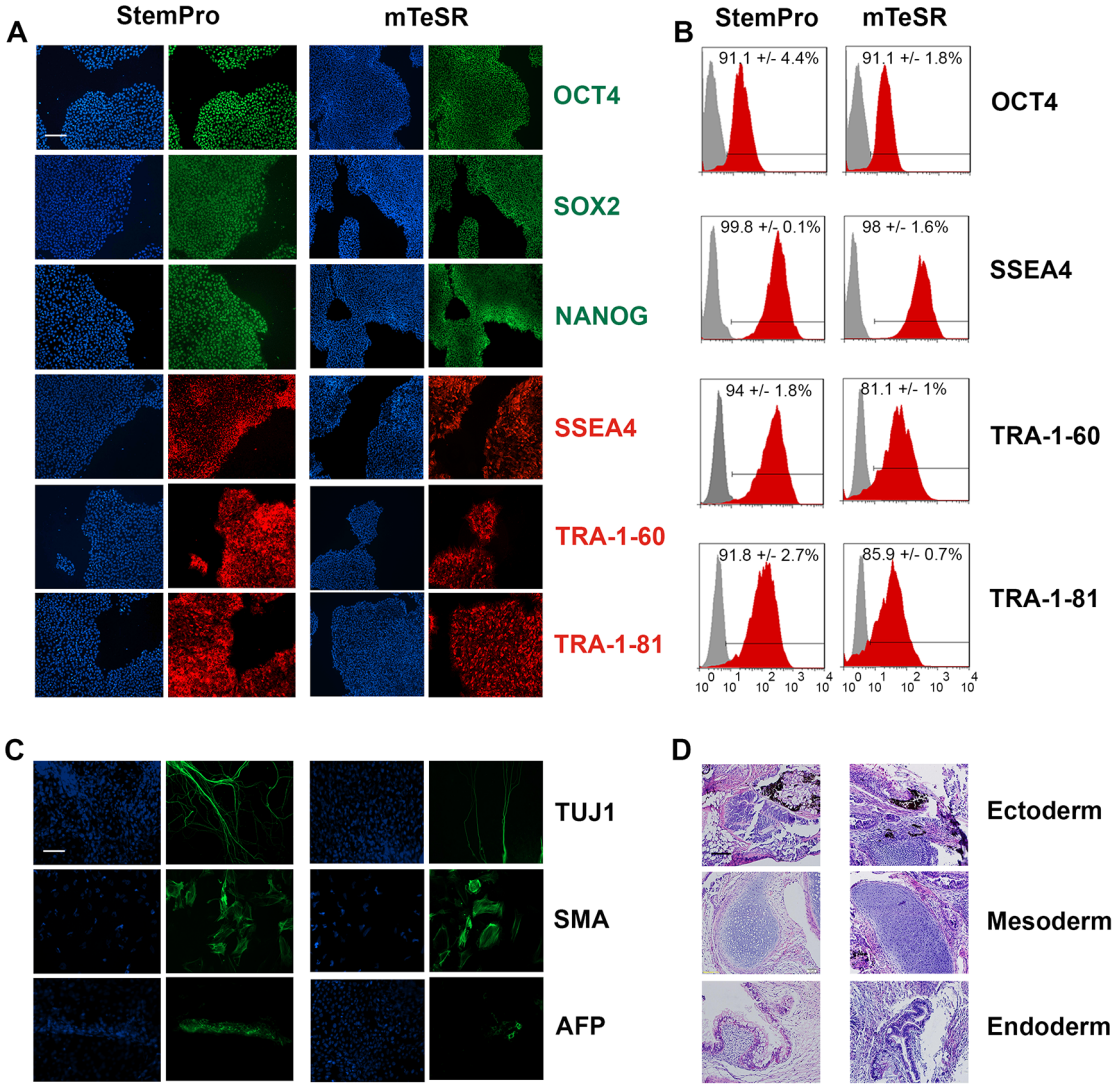
WA09 hESCs subcultured for over 25 passages using hypertonic citrate retain their stem cell characteristics. (A) Immunodetection of Oct4, Sox2 and Nanog antigens (green); SSEA-4, Tra-1-60 or Tra-1-81 antigens (red). Individual cell nuclei are visualized using DAPI (blue). Scale bar: 200 µM. (B) Flow cytometric analysis performed on cells cultured in either StemPro® or mTeSR™1 using antibodies that detect Oct4, SSEA-4, Tra-1-60, and Tra-1-81 antigens. Cells expressing each pluripotent antigen, detected using a specific antibody are illustrated in red. The isotype control used to detect non-specific binding is shown in gray. (C) Immunohistochemistry performed on embryoid bodies differentiated for 7 days in suspension culture and 7 days on gelatin-coated plates. Antibodies detecting Beta-III-Tubulin (TUJ1), Smooth Muscle Actin (SMA) and Alpha-Feto Protein (AFP) antigens are shown (green). Nuclear staining is shown using DAPI (blue). Scale bar: 200 µM. (D) Tissue sections of teratomas produced from undifferentiated hESCs contain cells from the indicated germ layers. Sections are shown counterstained with Hematoxylin and Eosin. Scale bar: 200 µm.

The ability of hPSCs to self-renew and differentiate into specific cell types is a fundamental characteristic that must ultimately be retained with the introduction of any new cultivation method. Therefore, to complete our assessment of reagent compatibility it was essential to determine if hPSCs, continuously passaged using the hypertonic citrate solution, could retain their pluripotency and maintain a normal G-banded karyotype. We evaluated WA09 hESCs cultured on Matrigel™ in either StemPro® at passage 31 (P31) or mTeSR™1 at P34. Both cell populations expressed the classic subset of nuclear and cell surface markers indicative of hPSC pluripotency: Oct4, Sox 2, Nanog, SSEA4, Tra-1-60 and Tra-1-81 (Fig. 5A). Flow cytometric analysis revealed that over 80% of the WA09 cells cultured in StemPro® (P36) and mTeSR™1 (P30) co-expressed Oct4, SSEA4, Tra-1-60 and Tra-1-81 (Fig. 5B). Additionally, WA09 hESCs subcultured in StemPro® (P35) and mTesR™1 (P40) were capable of forming embryoid bodies composed of cells expressing early markers of differentiation for ectoderm, mesoderm and endoderm (Fig. 5C). Moreover, hESCs cultivated in StemPro® and mTesR™1 (P>25) were injected into immune-compromised SCID/Beige mice. The cells generated multilineage teratomas, further illustrating their pluripotency *in vivo* (Fig. 5D). Finally, G-banded karyotype analysis was performed on three independent cultures of WA09 hESCs maintained exclusively in either StemPro® (P25) or mTeSR™1 (P27) and continuously passaged using the 570 mOsmol/kg citrate solution. Twenty G-banded metaphase cells were analyzed from each independent culture. All six samples (3 using mTeSR™1 and 3 using StemPro®) were normal based on this analysis.

### Characterization of additional hPSCs

Since different hPSC lines can respond differently to the same culture conditions, we characterized an additional hESC line and two independent iPSC lines for at least 30 passages using the 570 mOsmol/kg citrate solution. These lines were then evaluated for their ability to express markers of pluripotency, differentiate to cell types representative of all three germ layers and maintain a normal G-banded metaphase karyotype. Flow cytometric analysis revealed that all three lines expressed the classic subset of cell surface markers indicative of hPSC pluripotency (>80%) and were capable of producing embryoid bodies composed of cells expressing early markers of differentiation for ectoderm, mesoderm and endoderm (Table 2).

**Table 2:** Characterization of additional hPSC lines continuously passaged using the 1/kg citrate solution.

Cell Type	Total	G-Banded	SSEA4,	Ectoderm	Mesoderm	Endoderm
	Passages	Karyotype	Tra-1-60 Tra-1-81	(Tuj1)	(SMA)	(AFP)
**WA07**	40	Normal	>80%	Positive	Positive	Positive
**hiPSC18R**	30	Normal	>80%	Positive	Positive	Positive
**hiPSC19K**	30	Normal	>80%	Positive	Positive	Positive

## Discussion

Our search for an improved passaging method for hPSC cultivation was defined by a need to streamline and reduce the technical variability resulting in cell loss using existing adherent small- and large-scale hPSC cultivation processes. This is an important step in the translation of hPSC cultivation practices to clinical applications. The scale of hPSCs needed for different types of cell therapies varies widely depending on the targeted patient population. Small- and medium -scale applications are sufficient to cover most autologous cell therapies. Multi-layer flasks and microcarrier systems, designed for large-scale adherent culture, are currently being applied to hPSC cultivation for the production of master cell banks and allogeneic cell therapy applications.

Conventional manual and enzymatic methods used to subcultivate hPSCs inherently result in substantial cell loss due to cell trauma and death. A recently reported non-enzymatic method using EDTA works well for small-scale cultivation of hPSCs, however its use is not compatible with large-scale cultures where access is restricted and longer operating times are required to recover the cells. The rapid reattachment of EDTA-treated hPSCs cells back to their matrix after addition of fresh culture medium is mentioned by the author's in their original protocol and they state the need to rapidly remove the cells to avoid cell loss [3].

We initially defined and formulated a simple non-enzymatic cell dissociation reagent that gently and reproducibly dislodges adherent WA09 cells from their substrate as multicellular aggregates and promotes high post-detachment viability (97%±1%) over standard and extended treatment times up to twenty minutes. The composition of the final passaging formulation was unexpected: a hypertonic (570 mOsmol/kg) 1 mM sodium citrate solution. Sodium citrate is established as a mild chelating agent with a lower affinity for divalent cations than EDTA [10]. It promotes cell dissociation by binding the divalent cations present in the aqueous extracellular environment and intercellular space between cells. This disrupts molecules involved in maintaining cell adhesion such as calcium-dependent cadherins [11] and calcium- and magnesium-dependent integrins [12], [13]. The osmolality of the sodium citrate solution has a clear effect on the size of the detached cell aggregates. In an isotonic 1 mM solution of sodium citrate (270 mOsmol/kg), cell-cell dissociation is likely to occur simultaneously with cell-matrix detachment. Over an extended treatment time of 20 minutes, this results in the dissociation of the hESC colonies to very small cell aggregates and single cells. However, by incrementally increasing the osmolality of the 1 mM sodium citrate solution, cell volume incrementally decreases. This may decrease the stress on intercellular attachments, allowing cell-matrix detachment to occur more rapidly. At osmolalities near 570 mOsmol/kg, the percentage of very small aggregates and single cells is minimized and the cell aggregates produced attach quickly to fresh matrix, spread and form monolayer colonies which proliferate and reach confluence within 5 to 7 days.

Cell cultivation practices for hPSCs have often included acute treatment of cells with non-isotonic solutions, particularly in commonly utilized methods of cryopreservation and vitrification [14]. However, to confirm the continual acute use of a hypertonic citrate solution did not have an adverse effect on the quality of the hESC cultures, we assessed the effect of the 570 mOsmol/kg solution containing 1 mM sodium citrate on WA09 cells. Subcultivation of WA09 hESCs with the hypertonic citrate solution for 25 passages had no adverse effect on their G-banded karyotype or the expression of pluripotency markers Oct4, Nanog, SSEA4, Tra-1-60 or Tra-1-81. Similarly, these hESCs exhibited no change in their ability to form embryoid bodies or differentiate to cells expressing early markers of ectoderm, mesoderm and endoderm. Further analysis of their ability to form multi-lineage teratomas substantiated their continued differentiation potential.

To assess the ability of the hypertonic citrate solution to maintain the pluripotency of other hPSC lines, additional long-term cultivation experiments were performed on the hESC line, WA07, and two independent hiPSC lines generated from CD34+ cord blood cells. Characterization of the WA07, iPSC18R and iPSC19K lines after 30 passages demonstrated that these hPSC lines maintained a normal G-banded karyotype, expressed high levels of markers consistent with pluripotency and were able to differentiate to all three germ layers.

The important advances observed with the development of this new passaging method are the production of small multicellular aggregates that resist further dissociation over extended treatment times and the increase in recovery of cells from multilayer flasks after addition of fresh culture medium to halt the sequestration of divalent cations. Together, these features effectively shorten the time and labor required to scale the production of hPSCs without requiring an excessive number of passages that could affect the quality of the cells. Using conventional colony scraping and enzymatic methods, one can normally expect to passage most hPSCs every four to six days using a seeding ratio of 1∶3 to 1∶6, depending on the robustness of the culture medium utilized. Using the 1 mM 570 mOsm/kg hypertonic solution of sodium citrate, these passaging ratios often increase to a range of 1∶10 or higher.
